# Prevalence of transcription factors in ascomycete and basidiomycete fungi

**DOI:** 10.1186/1471-2164-15-214

**Published:** 2014-03-20

**Authors:** Richard B Todd, Miaomiao Zhou, Robin A Ohm, Hendrika ACF Leeggangers, Loek Visser, Ronald P de Vries

**Affiliations:** 1 Department of Plant Pathology, Kansas State University, 4024 Throckmorton Plant Sciences Center, Manhattan, KS 66506, USA; 2 Fungal Physiology, CBS-KNAW, Uppsalalaan 8, 3584 CT Utrecht, The Netherlands; 3 Microbiology & Kluyver Centre for Genomics of Industrial Fermentations, Utrecht University, Uppsalalaan 8, 3584 CT Utrecht, The Netherlands; 4 Current address: US Department of Energy (DOE) Joint Genome Institute (JGI), 2800 Mitchell Drive, Walnut Creek, CA 94598, USA; 5 Current address: Department of Plant Physiology, Wageningen University, Droevendaalsesteeg 1, 6708 PA, Wageningen, The Netherlands

**Keywords:** Transcription factor, Ascomycete, Basidiomycete, Gene regulation, Fungal genomes, Evolution, Zinc binuclear cluster, Zinc finger, DNA binding domain, Aspergillus

## Abstract

**Background:**

Gene regulation underlies fungal physiology and therefore is a major factor in fungal biodiversity. Analysis of genome sequences has revealed a large number of putative transcription factors in most fungal genomes. The presence of fungal orthologs for individual regulators has been analysed and appears to be highly variable with some regulators widely conserved and others showing narrow distribution. Although genome-scale transcription factor surveys have been performed before, no global study into the prevalence of specific regulators across the fungal kingdom has been presented.

**Results:**

In this study we have analysed the number of members for 37 regulator classes in 77 ascomycete and 31 basidiomycete fungal genomes and revealed significant differences between ascomycetes and basidiomycetes. In addition, we determined the presence of 64 regulators characterised in ascomycetes across these 108 genomes. This demonstrated that overall the highest presence of orthologs is in the filamentous ascomycetes. A significant number of regulators lacked orthologs in the ascomycete yeasts and the basidiomycetes. Conversely, of seven basidiomycete regulators included in the study, only one had orthologs in ascomycetes.

**Conclusions:**

This study demonstrates a significant difference in the regulatory repertoire of ascomycete and basidiomycete fungi, at the level of both regulator class and individual regulator. This suggests that the current regulatory systems of these fungi have been mainly developed after the two phyla diverged. Most regulators detected in both phyla are involved in central functions of fungal physiology and therefore were likely already present in the ancestor of the two phyla.

## Background

Gene regulation is of major importance for physiology of all organisms, and has been intensively studied in fungi. It ensures that the required genes are switched on and act under the circumstances they are needed, and allows fungi to respond to changing conditions. Thirty-seven classes of regulator proteins have been identified in fungi [1], such as C2H2 (PF00096) [2], Zn2Cys6 (PF00172) [3], Fungal Specific transcription factor domain (PF04082), bZIP (PF00170) [4], Histone-like transcription factors (PF00808) [5], HLH (PF00010) [6], HSF (PF00447) [7], Myb DNA-binding (PF00249) [8], TEA (PF01285) [9] and GATA (PF00320) [10]. They coordinate many cellular processes that control growth, survival or reproduction on particular substrates, under certain conditions, or in particular environmental niches. Therefore the presence or absence of specific regulators is intimately linked to fungal biodiversity.

Analysis of the first available eukaryotic genome indicated a likely diversity of regulators [11]. For example, a number of Zn2Cys6 regulators known in other fungi were absent in *Saccharomyces cerevisiae*[12]. Differences in regulatory protein repertoire were found particularly for this class of regulators, which was reduced in number in *Kluyveromyces lactis* compared with *S. cerevisiae*[13], and considerably expanded in *Aspergillus nidulans*[14] and *Magnaporthe oryzae*[15]. Furthermore, a range of functions for fungal Zn2Cys6 regulators lacking yeast orthologs have been described [16]. With an exponentially growing number of fungal genome sequences covering all branches of the fungal tree of life it is now possible to explore the regulatory diversity of fungi and trace the evolutionary origin of particular regulators. For several specific regulators their presence in sets of fungal genomes has been reported. The pentose catabolic pathway in *Aspergillus* is regulated by two transcriptional activators, XlnR and AraR [17]. While XlnR is present in nearly all tested filamentous ascomycetes, AraR appears to be restricted to the order of the Eurotiales that consists of *Aspergillus*, *Penicillium* and related genera. An even higher diversity was observed for regulators of galactose catabolism. A subset of the ascomycetes contains the regulator GalX that appears to be mainly involved in the oxidoreductive pathway in *Aspergillus niger* and *A. nidulans*[18,19]. In addition, *A. nidulans* contains a second regulator, GalR, that controls genes of the Leloir Pathway and for which orthologs were not detected in any of the other studied species [18]. The *A. nidulans* long chain fatty acid utilisation regulators FarA and FarB, which themselves are related in sequence, each have orthologs widely conserved in filamentous fungi and share a single common homolog in certain Hemiascomycetes [20]. In contrast, the short chain fatty acid utilisation regulator ScfA was very poorly conserved, with possible orthologs in *A. nidulans*, *Aspergillus fumigatus* and *Neurospora crassa*[20]. The *A. niger* extracellular protease regulator PrtT was identified only in certain Aspergilli [21].

Previous genome-wide studies of transcription factors have focussed on a single transcription factor family [12,22,23], a single species [24], or the relative representation of transcriptional regulator classes in the fungal kingdom [1,25]. The rapid growth in availability of fungal genomes, particularly those of Basidiomycetes, over the last few years has now yielded wider representation of genome sequence data across the various lineages of the fungal kingdom and provides the opportunity for a more detailed analysis of prevalence of transcription regulators across fungal genomes. In this paper we compared the distribution of regulator gene classes between currently available fungal genomes. We analysed the presence or absence of 64 characterised regulators in 108 fungal genomes to provide a comprehensive evaluation of fungal diversity with respect to regulatory systems. The regulators we have focussed on are all well characterised in at least one fungal species and represent a range of different physiological functions, including 21 regulators involved in development and/or morphology, 19 regulators involved in carbon metabolism, and 13 regulators involved in nitrogen and amino acid metabolism. Many of these regulators perform central functions in the organisms where they have been initially studied and therefore provide a good test set for the analysis of their prevalence and evolution in the fungal kingdom.

## Results

### Distribution of regulator classes throughout the fungal kingdom

**Figure 1:**
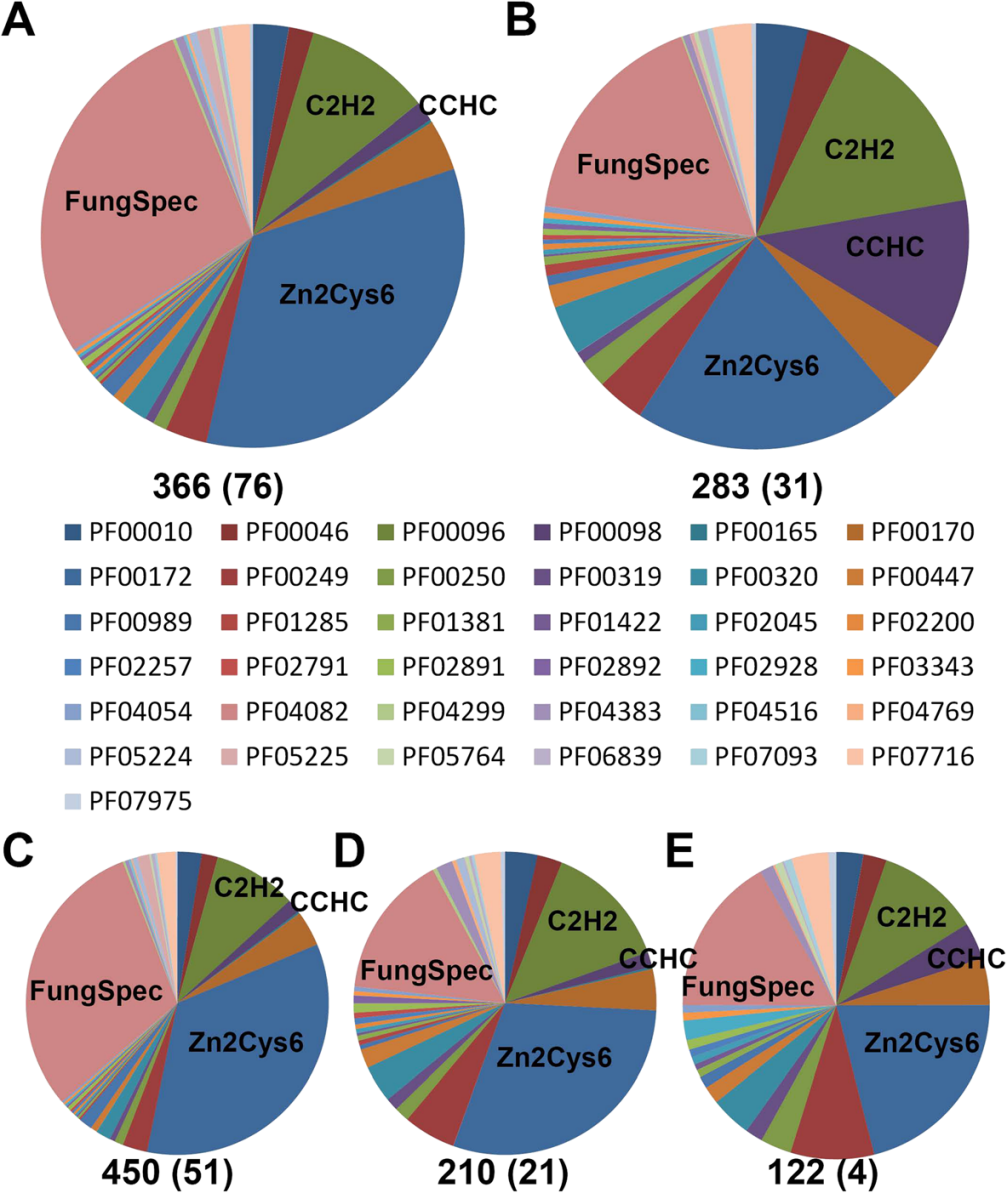
**Relative distribution of regulator PFAM family members in different fungal phyla. A**: ascomycetes, **B**: basidiomycetes, **C**: pezizomycotina; **D**: saccharomycotina; **E**: taphrinomycotina. The description of the PFAM families can be found in Additional file 3. The average number of transcription factors for each phylum is indicated underneath each pie chart. The number of genomes analyzed in each phylum or subphylum is indicated in parentheses.

To determine whether there are major differences in the relative number of regulators from different classes in the different fungal phyla, a PFAM analysis of the 37 known fungal transcription regulator-related PFAM domains [1] was performed on 77 ascomycete and 31 basidiomycete genomes (Additional file 1). A total of 36,636 putative transcription factors were identified (Additional file 2). Interesting differences in the relative number of regulators from different PFAM classes could be observed between the two phyla (Figure 1, Additional file 3). When comparing Ascomycota and Basidiomycota the main differences are a much larger expansion of the Zn2Cys6 domain family (PF000172) and the fungal specific transcription factor domain proteins (PF04082) in the Ascomycota, while in the Basidiomycota the C2H2 family (PF00096) and the CCHC zinc-finger family (PF00098) form a significantly higher percentage of the total number of regulators (Figure 1). This indicates that after these phyla split different regulatory strategies have developed based on different regulator classes. Within the Ascomycota, pezizomycetes contained the highest average amount (450) of putative regulators compared to saccharomycetes (210) and taphrinomycetes (122). Moreover, the Zn2Cys6 domain family and fungal specific transcription factor domain proteins in pezizomycotina were found in higher proportions than in the saccharomycotina and taphrinomycotina indicating the major expansion of these regulator classes occurred after divergence of the pezizomycotina from the other lineages. Unlike in the Basidiomycota, the lower abundance of these two families in the saccharomycotina and taphrinomycotina is not accompanied with a higher abundance of the C2H2 and CCHC families.

**Figure 2:**
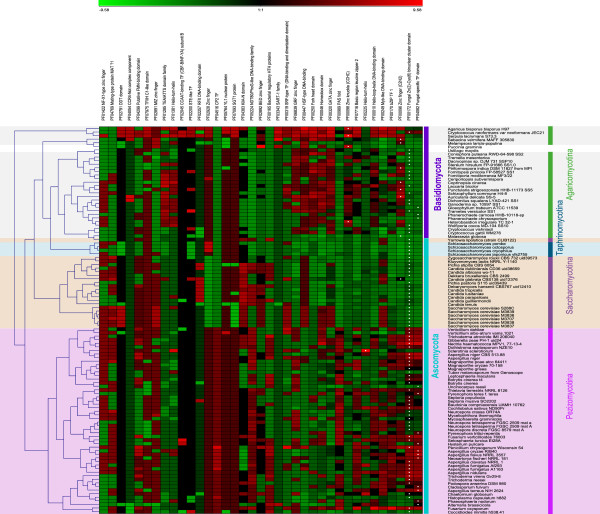
**Hierarchical clustering of fungal species by the abundance of regulators in PFAM families.** The difference between species in abundance of each PFAM family is shown. Values of presence and absence patterns were normalized by z-transformation across PFAM families and coloured so that green indicates the value is below the median for that PFAM family, whereas red indicates the value is higher than the median. The brighter the green, the lower the abundance across species, whereas the brighter the red, the higher the abundance across species. The largest PFAM class for each species is marked by the white dot in the corresponding colour square.

To compare the PFAM distribution of transcription factors between different fungal species, we used hierarchical clustering. The 36,636 regulators identified in the PFAM analysis were clustered, using OrthoMCL followed by manual curation, into 2,887 non-redundant orthologous groups (Additional file 4). These ortholog groups were then used to analyze the distribution of putative regulators among the PFAM families in fungi. A clear trend of regulator family distribution could be detected when species were clustered based on the transcription factor abundance pattern of the families (Figure 2). Interestingly, within Ascomycota only pezizomycotina species were clustered as one distinct group, the other major subdivisions, saccharomycotina and taphrinomycotina, were clustered within the Basidiomycota as two separate groups next to agaricomycotina. This indicates that after these phyla diverged, different regulator classes have been exploited in different lineages.

### Prevalence of specific regulators in fungal genomes

**Figure 3:**
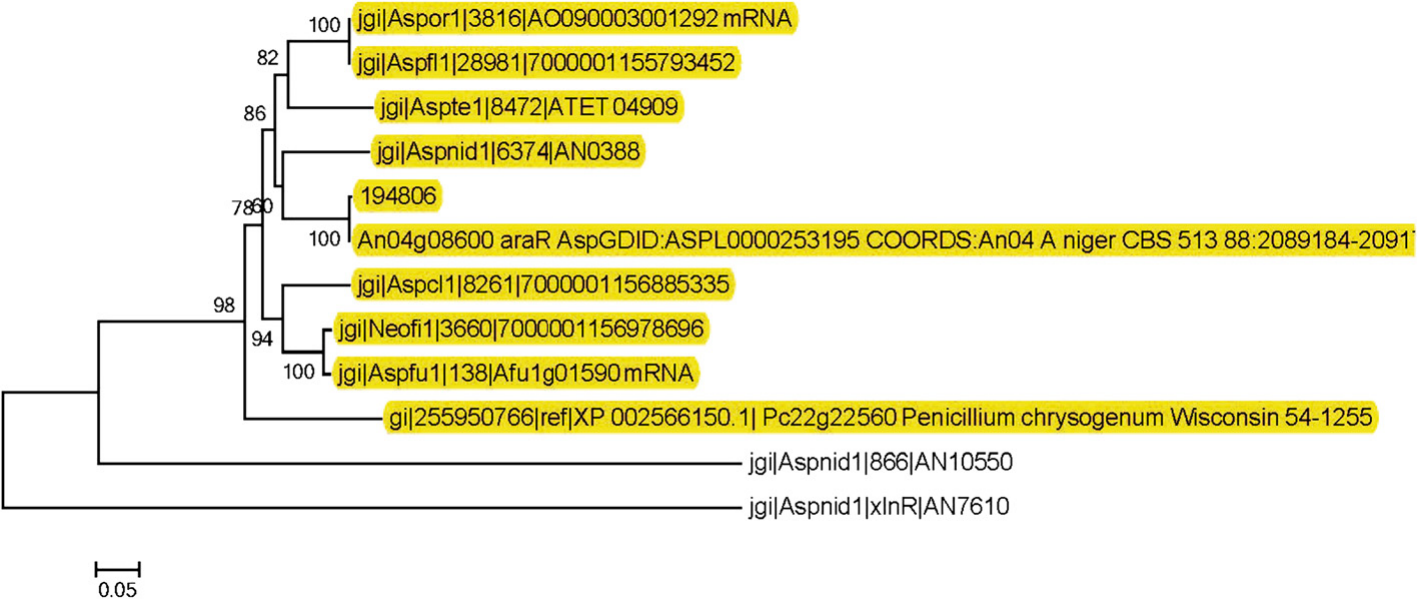
**Example of phylogenetic identification of false orthologs.** Neighbor-Joining tree of the AraR homologs using *Aspergillus nidulans* XlnR as an outgroup. The genes marked in yellow were maintained in the comparison. AN10550 was manually removed, as it clearly did not fall into the same cluster as the other genes. In fact, this gene is GalR, which is unique to *A. nidulans.* The gene identifiers can be found in Additional file 7.

A pilot study using bidirectional BlastP analysis to identify putative orthologs of a subset of chosen regulators was performed followed by manual curation to determine the parameters for automated analysis of the prevalence of regulators in fungal genomes (data not shown). This automated analysis was used to test for the presence or absence of orthologs of 64 regulators (Additional file 5) in the 108 genomes used for the PFAM distribution analysis above (Additional file 6, Additional file 7). A cut-off designated for identification of distant homologs [26] was applied throughout the survey in order to decrease the false negative rate caused by the highly divergent sequences of regulators. The results were then manually curated based on sequence alignments and phylogeny to remove false positives. An example is presented for AraR, where GalR was identified as a false positive (Figure 3).

Although the lowest number of orthologs was identified in the Basidiomycota, orthologs for five regulators involved in development and/or morphology (DopA, SteA, RlmA, MedA, Con7), the carbon catabolite repressor CreA, and the general expression activators HapB, HapC and HapE, are commonly found in basidiomycetes. Most of these regulators have general functions for fungal physiology, which explains their common distribution among fungi. Conversely, orthologs for six of the seven regulators from *Schizophyllum commune* (Fts3, Fts4, Hom1, Hom2, Gat1, C2H2) were only detected in basidiomycetes, while the other (WC2) also had orthologs in filamentous ascomycetes. Interestingly, no basidiomycete orthologs were detected for any of the transcriptional activators involved in plant biomass utilization (XlnR, AmyR, InuR, AraR, GalR, GalX, RhaR). All these regulators are members of the Zn2Cys6 class (Additional file 5), which is particularly expanded in ascomycetes compared to basidiomycetes (Figure 1). Indications for similar regulation systems related to plant biomass degradation have been found in transcriptomics studies of basidiomycetes [27-33]. However, the absence of orthologs for the ascomycete regulators suggests that these regulators have developed after the split from the ascomycetes, and the underlying molecular mechanisms may differ.

The overall low number of regulators for which an ortholog could be found in the basidiomycetes fits with the general PFAM distribution (see above) in which clear differences were found in the expansion of the different PFAM families between ascomycetes and basidiomycetes. This suggests a smaller regulatory repertoire in the ancestral fungus, which has undergone significant evolution since the basidiomycetes and ascomycetes separated.

The ascomycete yeast genomes also lack a significant number of the regulators, in particular those involved in plant biomass degradation and those involved in development. As most yeasts are not able to degrade plant biomass, nor go through developmental changes, this fits well with their physiology. Interestingly, there is a division into two groups with respect to the presence of CreA orthologs. *Saccharomyces* lacks this regulator, but instead has MIG1, which is the functional homolog of CreA, despite low sequence similarity. MIG1 orthologs were not found in any of the other tested fungi (Additional file 7).

The most diverse profiles can be seen for the filamentous ascomycetes. Regulators that are particularly poorly conserved in this group include two involved in development and/or morphology (AbaA, BrlA, mainly limited to Aspergilli and *Penicillium*), seven involved in carbon metabolism (AraR, GalX, GalR, ScfA, InuR, AlcR, AceII), one involved in nitrogen metabolism (AmdR), the iron homeostasis regulator SreA, the unfolded protein response regulator HacA and the aflatoxin biosynthesis regulator AflR, but many other differences can be observed. While the presence of some of these regulators appears to be evolutionarily related (present in nearly all species of a certain fungal clade) others are more dispersed through the ascomycete tree of life, suggesting that the regulator was present in their common ancestor but has been lost in specific species of different lineages.

Present in nearly all of the filamentous Ascomycetes (with a cut-off of three genomes missing the regulator) are eleven regulators involved in development and/or morphology (DopA, RosA, SteA, RlmA, MedA, DevR, Hsf2, Con7, StuA, WC2, VeA), four involved in carbon metabolism (FacB, FarB, AceI, AmdX), seven involved in nitrogen metabolism (UaY, LeuB, CpcA, NirA, AreB alpha, Nut1, NmrA), the His-Asp phosphorylation signalling regulator SrrA, the CCAAT-binding complex components HapB, HapC and HapE, the sulphur metabolic regulator MetR, and the penicillin biosynthesis regulator PenR2, providing a core set of transcriptional regulators that control most aspects of physiology.

For some transcription factors, multiple homologs were identified in the same species. In those cases where manual curation did not allow elimination of the additional copies, they were retained in the output data set (Additional file 3, Additional file 4, Additional file 6 and Additional file 7). We do not assume that the different copies will have the same function, although they are likely involved in similar processes. Functional analysis of these proteins will be needed to reveal their biological role.

## Discussion

Genomic studies of fungal transcription regulators have generally focused on a single transcription factor class in a particular species (e.g. [12,22,23]), or on the transcription factor complement within one species (e.g. [15,23]). However, analyses of transcription factor families have been conducted across a range of fungal genomes ( [1,25]). One study identified 37 PFAM families of transcription factors represented in 62 fungal genomes, and revealed the Zn2Cys6 zinc binuclear cluster and the fungal-specific transcription factor domain as the two largest fungal transcription factor classes [1]. Another study focussed on identification of transcription factors in 62 fungal genomes using the Fungal Transcription Factor Database (FTFD) phylogenomics pipeline, and determined the proportion of transcription factors amongst total predicted proteins [25]. Analysis of transcription factor family distribution revealed species-specific differences [25]. The aim of our study was to perform an inventory of the presence of regulators in the fungal kingdom, employing the expanded set of genome sequences that have become available in the last five years. Our analysis of the distribution of regulator classes indicated differential expansion of certain regulator types in ascomycetes and basidiomycetes, consistent with the development of many regulatory systems from a more limited ancestral set of regulators after the divergence of the two major fungal phyla. In ascomycetes, the Zn2Cys6 and fungal-specific domain regulators overwhelmingly predominated. This is consistent with previous identification of these two regulator classes as the most abundant fungal-specific regulators in a smaller set of mostly ascomycete fungal genomes [1]. The C2H2 zinc finger class comprised a smaller but major regulator class in the ascomycetes. Further analysis revealed a greater relative abundance of the Zn2Cys6 and fungal-specific domain regulators in the pezizomycotina than in the saccharomycotina and taphrinomycotina. In basidiomycetes, the C2H2 and CCHC classes showed a relative expansion and, with the Zn2Cys6 and fungal-specific domain regulators, comprise four similarly abundant major regulator classes. The differential expansion of regulator families in the fungal phyla and sub-phyla suggests that evolution of many regulators occurred after the divergence of these groups. The regulator distribution observed in our analysis showed some differences compared with the previously reported distributions in FTFD [25], most likely due to our expanded dataset. However, the abundance of Zn2Cys6 and C2H2 domain regulators reported in FTFD are compatible with our results.

As regulators play a major role in fungal physiology, their presence or absence may provide options and impose limitations on the natural habitat of fungal species. Analysis of the presence of individual transcription factors demonstrated that regulators with a central role in fungal physiology are most commonly found throughout the fungal tree of life, while regulators with more specific roles are less commonly present. This makes sense, as the loss of central regulators is likely to cause a significant competitive disadvantage for a species, unless transcriptional network functions are maintained by transcriptional rewiring. In contrast, the more specific regulators and the regulons they control will only be essential or advantageous in particular habitats.

As the characterised query regulators for our transcription factor presence/absence analysis were mainly from ascomycete fungi, it is not surprising that a relatively low number of orthologs was found in the genomes from basidiomycetes. Regulation of gene expression is poorly studied in these fungi compared with ascomycetes, but our data suggests that many of the regulatory systems have developed after the split of these two phyla. Interestingly, differences in the presence and absence of regulators were also found in closely related species. While it cannot be fully excluded that this can be due to gaps in the genome sequence or errors in gene annotation of specific genomes, this does suggest that changes in the regulatory systems have also occurred more recently. Examples of this are the GalX/GalR system for regulation of galactose catabolism [18] and the protease regulator PrtT [21], which was shown to differ significantly between the Aspergilli, and the specific presence of the cellulose regulator AceII [34].

While the absence of a particular regulator may accompany loss of an entire regulon and therefore an altered metabolic or developmental capability, its absence could indicate transcriptional rewiring of the regulatory mechanism. Conversely, presence of a regulator ortholog also does not necessarily indicate conserved function. Recent studies have shown that transcription regulatory mechanisms can display considerable plasticity across species. For some regulons the regulator components are conserved but exhibit functional reassignment and rewired circuitry, resulting in rearrangements of transcriptional networks [35]. Other regulons share a conserved overall strategy but include additional regulator components to integrate additional regulatory signals, or show transfer of regulation from one regulator to another, or rewiring via evolution of combinatorial interactions between transcription factors [36-38]. The array of transcriptional rewiring possibilities indicates that while the absence of a particular transcription factor ortholog suggests regulatory differences or the loss of regulons, the presence of orthologs may, but does not necessarily indicate conserved function. Therefore functional analysis is required to determine the role of each transcription factor in each species.

## Conclusion

We have conducted an inventory of the thirty-seven PFAM transcription factor classes across 108 genomes of the two major fungal phyla and shown differential expansion of transcription regulator classes between the ascomycetes and the basidiomycetes, with the largest expansion of Zn2Cys6 and fungal-specific domain regulators in the pezizomycotina. We also analyzed the presence profiles for 64 known regulators in these 108 genomes and found that regulators with central functions in fungal physiology were more commonly present than those with more specialised roles. The increasing number of fungal genome sequences and functional analyses will provide better insight in the evolution of regulatory systems and in particular the 1000 fungal genome project [39] will add to this as it aims to cover the breadth of the fungal kingdom.

## Methods

### Pilot experiment

A bidirectional BlastP analysis was performed using as query the amino acid sequences of 48 selected regulators. To manually curate the results, alignments of the hits for each query regulator were performed using MUSCLE [40] and manually corrected in MEGA4 [41]. Phylogenetic trees were generated with MEGA4 using three algorithims: Maximum Parsimony, neighbor joining and minimum evolution. The stability of the clades was tested with 1000 bootstrap replicates. The results of the manual curation were used to define the parameters for the automated analysis of a larger set of genomes.

### Large-scale genome study

108 completed fungal genomes were extracted from the JGI fungal program [42], Broad Institute of Harvard and MIT [43], AspGD [44,45] and NCBI genbank [46] (data version March 2013). Pfam-A HMM model was downloaded from the Pfam database [47]. Regulator-related domains were identified in each fungal genome in HMMerv. 3.0 [48] using the trust cutoff. Genome scale protein ortholog clusters were detected according to [49], using inflation factor 1, E-value cutoff 1E-3, percentage match cutoff 60% as for identification of distant homologs [26]. The all-vs-all BlastP search required by OrthoMCL was carried out in a grid of 500 computers by parallel fashion. The orthologs clusters were then curated manually by expert knowledge and literature search. Manual curation was aided by aligning the amino acid sequences of the hits for each query together with a suitable outgroup by MAFFT [50,51], after which neighbor joining trees were generated using MEGA5 with 1000 bootstraps. Genes that were clearly separated from the query branch in the trees were removed from the results. An example of this is given for AraR in Figure 3. Putative regulators containing more than one PFAM domain were assigned to the cluster based on the number of copies of domains found and/or the length of aligned area to the domain. PFAM families in 108 genomes were clustered by mismatch distance using Genesis [52]. The dendrogram was drawn by the complete linkage method using Genesis. A z-transformation of data was performed across families in order to generate the color scheme for visualization.

### Availability of supporting data

The data sets supporting the results of this article are included within the article and its additional files, or are available in the Dryad Digital Repository [53].

## Competing interests

The authors declare that they have no competing interests.

## Authors’ contributions

RAO performed the genome mining for the pilot experiment, while MZ performed the large scale genome mining. HACFL and LV performed manual curation on the pilot experiment. RBT and RPdV designed the study. RBT, RPdV and MZ wrote the manuscript. All authors contributed to the interpretation of the data. RBT and MZ contributed equally. All authors read and approved the final manuscript.

## Supplementary Material

Additional file 1 **Fungal genomes used in this study.** Species, genome sequence file name, phylogenetic division, source of genome sequence data, and reference [54-104]. Click here for file

Additional file 2 Putative regulators identified by Pfam in 108 fungal genomes. Click here for file

Additional file 3 **Distribution of numbers of putative regulator encoding genes per PFAM family in different fungi.** The number of regulators in each PFAM class found in each of the 108 genomes analysed. Click here for file

Additional file 4 **The orthologous clusters of putative regulators in 108 fungi.** Gene ID of all 36,636 putative transcriptional factors clustered in 2,887 non-redundant orthologous groups. Click here for file

Additional file 5 **Regulators used in this study Transcription factor, family, process/function, species, and reference [**[9]**,**[17]**,**[18]**,**[20]**,**[21]**,**[34]**,**[105]**-**[152]**].** Click here for file

Additional file 6 **Presence of homologs of the 64 selected regulators in 108 fungal genomes.** Presence or absence of homologs of each regulator are tabulated for the 108 fungal genomes analysed. Click here for file

Additional file 7 **Gene IDs of the homologs of the 64 regulators.** Gene IDs are displayed as indicated in the genome downloads. In the case of mutiple homologs, the gene ID’s are in the same cell separated by a comma. Click here for file
