# Supplementary material for: Prevalence of transcription factors in ascomycete and basidiomycete fungi
Source: BMC Genomics. 2014 Mar 20;15:214. doi: 10.1186/1471-2164-15-214 (PMC3998117; doi:10.1186/1471-2164-15-214)
Supplement: Additional file 1 — Fungal genomes used in this study. Species, genome sequence file name, phylogenetic division, source of genome sequence data, and reference [54-104]. [file 1471-2164-15-214-S1.docx]

**Additional file 1. Fungal genomes used in this study.**

| **Species** | **File name** | **Division** | **Source** | **References** |
| --- | --- | --- | --- | --- |
| *Alternaria brassicicola* | Alternaria_brassicicola_proteins.fasta | Ascomycota | JGI | [54] |
| *Baudoinia compniacensis* UAMH 10762 | Bauco1_GeneCatalog_proteins_20110511.aa.fasta | Ascomycota | JGI | [54] |
| *Cladosporium fulvum* v1.0 | Clafu1_GeneCatalog_proteins_20110826.aa.fasta | Ascomycota | JGI | [55] |
| *Cochliobolus sativus* ND90Pr | Cocsa1_GeneCatalog_proteins_20110610.aa.fasta | Ascomycota | JGI | [54] |
| *Dothistroma septosporum* NZE10 v1.0 | Dotse1_GeneCatalog_proteins_20100818.aa.fasta | Ascomycota | JGI | [55] |
| *Hysterium pulicare* | Hyspu1_GeneCatalog_proteins_20110209.aa.fasta | Ascomycota | JGI | [54] |
| *Leptosphaeria maculans* | Lepmu1_GeneCatalog_proteins_20110301.aa.fasta | Ascomycota | JGI | [54] |
| *Mycosphaerella graminicola* | Mgraminicolav2.FilteredModels1.proteins.fasta | Ascomycota | JGI | [56] |
| *Phaeosphaeria nodorum* | phaeosphaeria_nodorum_1_proteins.fasta | Ascomycota | Broad | [57] |
| *Pyrenophora teres f. teres* | Pyrtt1_GeneCatalog_proteins_20110408.aa.fasta | Ascomycota | JGI | [54] |
| *Pyrenophora tritici-repentis* | Pyrenophora_tritici_repentis_proteins.fasta | Ascomycota | JGI | [54] |
| *Septoria musiva* SO2202 v1.0 = *Mycosphaerella populorum* SO2202 | Sepmu1_GeneCatalog_proteins_20100915.aa.fasta | Ascomycota | JGI | [54] |
| *Septoria populicola* v1.0 = *Mycosphaerella populicola* PO2.02b | Seppo1_GeneCatalog_proteins_20110720.aa.fasta | Ascomycota | JGI | [54] |
| *Setosphaeria turcica* Et28A | Settu1_GeneCatalog_proteins_20110305.aa.fasta | Ascomycota | JGI | [54] |
| *Coccidioides immitis* h538.4 1 | coccidioides_immitis_h538.4_1_proteins.fasta | Ascomycota | Broad | [58] |
| *Aspergillus clavatus* NRRL 1 | Aspcl1_GeneCatalog_proteins_20120815.aa.fasta | Ascomycota | AspGD | [59] |
| *Aspergillus flavus* NRRL 3557 | Aspfl1_GeneCatalog_proteins_20121018.aa.fasta | Ascomycota | AspGD | [60] |
| *Aspergillus fumigatus* A1163 | A_fumigatus_A1163_orf_trans_all.fasta | Ascomycota | AspGD | [59] |
| *Aspergillus fumigatus* Af293 | Aspfu1_GeneCatalog_proteins_20120808.aa.fasta | Ascomycota | AspGD | [61] |
| *Aspergillus nidulans* FGSC A4 | Aspnid1_GeneCatalog_proteins_20110130.aa.fasta | Ascomycota | AspGD | [62] |
| *Aspergillus niger* ATCC 1015 | Aspergillus_niger_v3_FilteredModels_proteins.fasta | Ascomycota | AspGD | [63] |
| *Aspergillus niger* CBS 513.88 | A_niger_CBS_513_88_version_s01-m04-r27_orf_trans_all.fasta | Ascomycota | AspGD | [63] |
| *Aspergillus oryzae* RIB40 | Aspor1_GeneCatalog_proteins_20120808.aa.fasta | Ascomycota | AspGD | [64] |
| *Aspergillus terreus* NIH 2624 | Aspte1_GeneCatalog_proteins_20120919.aa.fasta | Ascomycota | AspGD | [59] |
| *Histoplasma capsulatum* h88 2 | histoplasma_capsulatum_h88_2_proteins.fasta | Ascomycota | Broad | [58] |
| *Neosartorya fischeri* NRRL 181 | Neofi1_GeneCatalog_proteins_20120815.aa.fasta | Ascomycota | JGI | [59] |
| *Penicillium rubens* Wisconsin 54 | Penicillium_chrysogenum_Wisconsin_54_1255_uid39879.faa | Ascomycota | GenBank | [65] |
| *Uncinocarpus reesii* | uncinocarpus_reesii_2_proteins.fasta | Ascomycota | Broad | [58] |
| *Botrytis cinerea* t4 | botrytis_cinerea__t4__1_proteins.fasta | Ascomycota | GenBank | [66] |
| *Botrytis cinerea* v1.0 | Botci1_GeneCatalog_proteins_20110903.aa.fasta | Ascomycota | JGI | [66] |
| *Sclerotinia sclerotiorum* v1.0 | Sclsc1_GeneCatalog_proteins_20110903.aa.fasta | Ascomycota | JGI | [66] |
| *Tuber melanosporum* from Genoscope | Tubme1_GeneCatalog_proteins_20111120.aa.fasta | Ascomycota | JGI | [67] |
| *Candida albicans* wo-1 1 | candida_albicans_wo-1_1_proteins.fasta | Ascomycota | Broad | [68] |
| *Candida dubliniensis* CD36 uid38659 | Candida_dubliniensis_CD36_uid38659.faa | Ascomycota | Broad | [68] |
| *Candida glabrata* CBS138 uid12376 | Candida_glabrata_CBS138_uid12376.faa | Ascomycota | Broad | [68] |
| *Candida guilliermondii* | candida_guilliermondii_1_proteins.fasta | Ascomycota | Broad | [68] |
| *Candida lusitaniae* | candida_lusitaniae_1_proteins.fasta | Ascomycota | Broad | [68] |
| *Candida parapsilosis* | candida_parapsilosis_1_proteins.fasta | Ascomycota | Broad | [68] |
| *Candida tenuis* | Cante1_filtered_proteins_v1_1.fasta | Ascomycota | Broad | [68] |
| *Candida tropicalis* | candida_tropicalis_3_proteins.fasta | Ascomycota | Broad | [68] |
| *Debaryomyces hansenii* CBS767 uid12410 | Debaryomyces_hansenii_CBS767_uid12410.faa | Ascomycota | GenBank | [69] |
| *Dekkera bruxellensis* CBS 2499 | Dekbr2_GeneCatalog_proteins_20120306.aa.fasta | Ascomycota | JGI | [70] |
| *Kluyveromyces lactis* NRRL Y-1140 | Kluyveromyces_lactis_NRRL_Y-1140_uid12377.faa | Ascomycota | GenBank | [69] |
| *Pichia pastoris* S115 uid39439 | Pichia_pastoris_GS115_uid39439.faa | Ascomycota | GenBank | [68] |
| *Pichia stipitis* CBS 6054 | Pstipitisv2.FilteredModels1.proteins | Ascomycota | JGI | [68] |
| *Saccharomyces cerevisiae* M3707 v1.0 | SacceM3707_1_GeneCatalog_proteins_20120712.aa.fasta | Ascomycota | JGI | [71] |
| *Saccharomyces cerevisiae* M3836 v1.0 | SacceM3836_1_GeneCatalog_proteins_20120712.aa.fasta | Ascomycota | JGI | [71] |
| *Saccharomyces cerevisiae* M3837 v1.0 | SacceM3837_1_GeneCatalog_proteins_20120713.aa.fasta | Ascomycota | JGI | [71] |
| *Saccharomyces cerevisiae* M3838 v1.0 | SacceM3838_1_GeneCatalog_proteins_20120714.aa.fasta | Ascomycota | JGI | [71] |
| *Saccharomyces cerevisiae* M3839 v1.0 | SacceM3839_1_GeneCatalog_proteins_20120714.aa.fasta | Ascomycota | JGI | [71] |
| *Saccharomyces cerevisiae* S288C | Sacce1_GeneCatalog_proteins_20101210.aa.fasta | Ascomycota | JGI | [72] |
| *Yarrowia lipolytica* (strain CLIB122) | Yarli1_GeneCatalog_proteins_20130122.aa.fasta | Ascomycota | JGI | [69] |
| *Zygosaccharomyces rouxii* CBS 732 uid39573 | Zygosaccharomyces_rouxii_CBS_732_uid39573.faa | Ascomycota | GenBank | [73] |
| *Schizosaccharomyces cryophilus* | schizosaccharomyces_cryophilus_4_proteins.fasta | Ascomycota | Broad | [74] |
| *Schizosaccharomyces octosporus* | schizosaccharomyces_octosporus_6_proteins.fasta | Ascomycota | Broad | [74] |
| *Schizosaccharomyces japonicus* yfs275 5 | schizosaccharomyces_japonicus_yfs275_5_proteins.fasta | Ascomycota | Broad | [74] |
| *Chaetomium globosum* v1.0 | Chagl_1_GeneModels_BroadGeneModels_aa.fasta | Ascomycota | Broad | [75] |
| *Fusarium oxysporum* v1.0 | Fusox1_GeneCatalog_proteins_20110522.aa.fasta | Ascomycota | JGI | [76] |
| *Fusarium verticillioides* 7600 3 | fusarium_verticillioides_7600_3_proteins.fasta | Ascomycota | Broad | [76] |
| *Gibberella zeae* PH-1 uid24 | Gibberella_zeae_PH-1_uid243.faa | Ascomycota | GenBank | [77] |
| *Magnaporthe grisea* 70-5 v1.0 | Maggr1_GeneCatalog_proteins_20110524.aa.fasta | Ascomycota | JGI | [78] |
| *Magnaporthe oryzae* 70-15 8 | magnaporthe_oryzae_70-15_8_proteins.fasta | Ascomycota | Broad | [78] |
| *Magnaporthe poae* atcc 64411 | magnaporthe_poae_atcc_64411_1_proteins.fasta | Ascomycota | Broad | [79] |
| *Myceliophthora thermophila* (*Sporotrichum thermophile*) v2.0 | Spoth2_GeneCatalog_proteins_20101221.aa.fasta | Ascomycota | JGI | [80] |
| *Nectria haematococca* MPV1, 77-13-4 | Necha2_best_proteins.fasta | Ascomycota | JGI | [81] |
| *Neurospora crassa* OR74A | Neurospora_crassa.proteins.fasta | Ascomycota | JGI | [82] |
| *Neurospora discreta* FGSC 8579 matA | Ndiscreta.FilteredModels2.proteins.fasta | Ascomycota | JGI | [83] |
| *Neurospora tetrasperma* FGSC 2509 mat a | Ntetrasperma_mata.FilteredModels1.proteins.fasta | Ascomycota | JGI | [84] |
| *Neurospora tetrasperma* FGSC 2509 mat A | N.tetrasperma_matA_v2_FilteredModels.proteins.fasta | Ascomycota | JGI | [84] |
| *Podospora anserina* DSM 980 | Podospora_anserina_DSM_980_uid29799.faa | Ascomycota | GenBank | [85] |
| *Thielavia terrestris* NRRL 8126 | Thite2_GeneCatalog_proteins_20101221.aa.fasta | Ascomycota | JGI | [80] |
| *Trichoderma atroviride* IMI 206040 | Tatroviridev2_FrozenGeneCatalog_20100319.proteins.fasta | Ascomycota | JGI | [86] |
| *Trichoderma reesei* | TreeseiV2_FilteredModelsv2.0.proteins.fasta | Ascomycota | JGI | [87] |
| *Trichoderma virens* Gv29-8 | Tvirens_v2.FrozenGeneCatalog_20100318.proteins.fasta | Ascomycota | JGI | [86] |
| *Verticillium albo-atrum* VaMs.102 1 | verticillium_albo-atrum_vams.102_1_proteins.fasta | Ascomycota | Broad | [86] |
| *Verticillium dahliae* VdLs17 v1.0 | Verda1_GeneCatalog_proteins_20110524.aa.fasta | Ascomycota | Broad | [86] |
| *Schizosaccharomyces pombe* | Schpo1_GeneCatalog_proteins_20130111.aa.fasta | Ascomycota | GenBank | [88] |
| *Agaricus bisporus* var *bisporus* H97 | Abisporus_varbisporusH97.v2.FilteredModels3.proteins.fasta | Basidiomycota | JGI | [89] |
| *Auricularia delicata* SS-5 | Aurde1_GeneCatalog_proteins_20110213.aa.fasta | Basidiomycota | JGI | [90] |
| *Ceriporiopsis subvermispora* B | Ceriporiopsis_subvermispora.proteins.fasta | Basidiomycota | JGI | [91] |
| *Coniophora puteana* RWD-64-598 SS2 | Conpu1_GeneCatalog_proteins_20101116.aa.fasta | Basidiomycota | JGI | [90] |
| *Coprinopsis cinerea* | Coprinopsis_cinerea.proteins.fasta | Basidiomycota | JGI | [92] |
| *Cryptococcus neoformans* var *neoformans* JEC21 | Cryne_JEC21_1_GeneCatalog_proteins_20120305.aa.fasta | Basidiomycota | JGI | [93] |
| *Cryptococcus vishniacii* v1.0 | Cryvi1_GeneCatalog_proteins_20130104.aa.fasta | Basidiomycota | JGI | [93] |
| *Dacryopinax* sp. DJM 731 SSP1 v1.0 | Dacsp1_GeneCatalog_proteins_20110131.aa.fasta | Basidiomycota | JGI | [90] |
| *Dichomitus squalens* LYAD-421 SS1 | Dicsq1_GeneCatalog_proteins_20101110.aa.fasta | Basidiomycota | JGI | [90] |
| *Fomitiporia mediterranea* MF3/22 | Fomme1_GeneCatalog_proteins_20101122.aa.fasta | Basidiomycota | JGI | [90] |
| *Fomitopsis pinicola* FP-58527 SS1 | Fompi3_GeneCatalog_proteins_20120705.aa.fasta | Basidiomycota | JGI | [90] |
| *Ganoderma* sp. 10597 SS1 | Gansp1_GeneCatalog_proteins_20110326.aa.fasta | Basidiomycota | JGI | [94] |
| *Gloeophyllum trabeum* ATCC 11539 | Glotr1_1_GeneCatalog_proteins_20100921.aa.fasta | Basidiomycota | JGI | [90] |
| *Heterobasidion irregulare* TC 32-1 | Hannosum_v2.FilteredModels1.proteins.fasta | Basidiomycota | JGI | [95] |
| *Laccaria bicolor* v2.0 | Lacbi2_GeneCatalog_proteins_20110203.aa.fasta | Basidiomycota | JGI | [96] |
| *Phanerochaete carnosa* HHB-10118-sp | Phaca1_GeneCatalog_proteins_20101213.aa.fasta | Basidiomycota | JGI | [97] |
| *Phanerochaete chrysosporium* | BestModels2.1.proteins | Basidiomycota | JGI | [98] |
| *Piriformospora indica* DSM 11827 from MPI | Pirin1_GeneCatalog_proteins_20111203.aa.fasta | Basidiomycota | JGI | [99] |
| *Punctularia strigosozonata* HHB-11173 SS5 | Punst1_GeneCatalog_proteins_20101026.aa.fasta | Basidiomycota | JGI | [90] |
| *Schizophyllum commune* H4-8 | Schco2_GeneCatalog_proteins_20110923.aa.fasta | Basidiomycota | JGI | [100] |
| *Sebacina vermifera* MAFF 305830 | Sebve1_GeneCatalog_proteins_20120626.aa.fasta | Basidiomycota | JGI | [99] |
| *Serpula lacrymans* S7.3 | Serpula_lacrymans_S7_3_v2.proteins.fasta | Basidiomycota | JGI | [101] |
| *Stereum hirsutum* FP-91666 SS1 v1.0 | Stehi1_GeneCatalog_proteins_20101026.aa.fasta | Basidiomycota | JGI | [90] |
| *Trametes versicolor* SS1 | Trave1_GeneCatalog_proteins_20101111.aa.fasta | Basidiomycota | JGI | [90] |
| *Tremella mesenterica* | Treme1_best_proteins.fasta | Basidiomycota | JGI | [90] |
| *Wolfiporia cocos* MD-104 SS10 | Wolco1_GeneCatalog_proteins_20100915.aa.fasta | Basidiomycota | JGI | [90] |
| *Melampsora laricis-populina* v1.0 | Mlaricis_populina.FilteredModels3.aa.fasta | Basidiomycota | JGI | [101] |
| *Puccinia graminis* | Puccinia_graminis.proteins.fasta | Basidiomycota | JGI | [101] |
| *Cryptococcus gattii* WM276 | Cryptococcus_gattii_WM276.faa | Basidiomycota | Broad | [102] |
| *Malassezia globosa* | Malassezia_globosa.proteins.fasta | Basidiomycota | JGI | [103] |
| *Ustilago maydis* | Ustilago_maydis.proteins.fasta | Basidiomycota | JGI | [104] |
