# Supplementary material for: Prevalence of transcription factors in ascomycete and basidiomycete fungi
Source: BMC Genomics. 2014 Mar 20;15:214. doi: 10.1186/1471-2164-15-214 (PMC3998117; doi:10.1186/1471-2164-15-214)
Supplement: Additional file 5 — Regulators used in this study Transcription factor, family, process/function, species, and reference [[9],[17],[18],[20],[21],[34],[105]-[152]]. [file 1471-2164-15-214-S5.docx]

**Additional file 5. Regulators used in this study.**

| **Transcription factor** | **Family** | **Process/Function** | **Species** | **Reference** |
| --- | --- | --- | --- | --- |
| **Development & morphology** | | | | |
| AbaA | TEA/ATTS | Terminal stages of asexual development | *Aspergillus nidulans* | [9] |
| BrlA | C2H2 | Asexual development | *Aspergillus nidulans* | [105] |
| C2H2 | C2H2 | Primordium development | *Schizophyllum commune* | [106] |
| Con7 | C2H2 | Disease-related morphogenesis | *Magnaporthe oryzae* | [107] |
| DevR | bHLH | Conidiophore development | *Aspergillus nidulans* | [108] |
| DopA | Leucine zipper | Cellular morphogenesis | *Aspergillus nidulans* | [109] |
| FlbD | Myb-like | Asexual development | *Aspergillus nidulans* | [110] |
| Fst3 | Zn2Cys6 | Mushroom development | *Schizophyllum commune* | [100] |
| Fst4 | Zn2Cys6 | Mushroom formation | *Schizophyllum commune* | [100] |
| Gat1 | GATA | Mushroom development | *Schizophyllum commune* | [106] |
| Hom1 | Homeodomain | Mushroom development | *Schizophyllum commune* | [106] |
| Hom2 | Homeodomain | Mushroom formation | *Schizophyllum commune* | [106] |
| Hsf2 | HSF | Asexual development | *Aspergillus parasiticus* | [111] |
| MedA |  | Sexual and asexual development | *Aspergillus nidulans* | [112] |
| NsdD | GATA | Sexual development | *Aspergillus nidulans* | [113] |
| RlmA | SRF | Cell wall reinforcement in response to cell wall stress | *Aspergillus niger* | [114] |
| RosA | Zn2Cys6 | Sexual development | *Aspergillus fumigatus* | [115] |
| SteA | C2H2 | Sexual reproduction | *Aspergillus nidulans* | [116] |
| StuA | APSES | Sexual and asexual development, Morphology | *Aspergillus nidulans* | [117] |
| VeA | Velvet | Sexual and asexual development | *Aspergillus nidulans* | [118] |
| WC2 | GATA | Blue light receptor, initiation of mushroom formation | *Schizophyllum commune* | [119] |
| **Carbon metabolism** | | | | |
| AceI | C2H2 | Repressor of xylanase and cellulase genes | *Trichoderma reesei* | [120] |
| AceII | Zn2Cys6 | Expression of xylanase and cellulase genes | *Trichoderma reesei* | [34] |
| AlcR | Zn2Cys6 | Ethanol utilization | *Aspergillus nidulans* | [121] |
| AmdA | C2H2 | Acetate induction of *amdS* and *aciA* genes/acetamide and formate utilization | *Aspergillus nidulans* | [122] |
| AmdX | C2H2 | Activation of *amdS* | *Aspergillus nidulans* | [123] |
| AmyR | Zn2Cys6 + Fung Trans | Utilization of maltose and starch | *Aspergillus nidulans* | [124] |
| AraR | Zn2Cys6 | Arabinose repression | *Aspergillus niger* | [17] |
| CreA | C2H2 | Carbon catabolite repression | *Aspergillus nidulans* | [125] |
| FacB | Zn2Cys6 + Fung Trans | Acetate utilization | *Aspergillus niger* | [126] |
| FarA | Zn2Cys6 + Fung Trans | Fatty acid utilization | *Aspergillus nidulans* | [20] |
| FarB | Zn2Cys6 + Fung Trans | Fatty acid utilization | *Aspergillus nidulans* | [20] |
| GalR | Zn2Cys6 + Fung Trans | Activation of galactose catabolism genes | *Aspergillus nidulans* | [18] |
| GalX | Zn2Cys6 + Fung Trans | Activation of galactose catabolism genes | *Aspergillus nidulans* | [18] |
| InuR | Zn2Cys6 + Fung Trans | Inducing inulinolytic genes | *Aspergillus niger* | [127] |
| MIG1 | C2H2 | Glucose repression | *Saccharomyces cerevisiae* | [128] |
| QutA | Zn2Cys6 + Fung Trans | Quinate utilization | *Aspergillus nidulans* | [129] |
| RhaR | Zn2Cys6 + Fung Trans | L-rhamnose release and catabolism | *Aspergillus niger* | [130] |
| ScfA | Zn2Cys6 + Fung Trans | Short chain fatty acid utilization | *Aspergillus nidulans* | [20] |
| XlnR | Zn2Cys6 + Fung Trans | Activation of xylanolytic enzymes | *Aspergillus niger* | [131] |
| **Nitrogen and amino acid metabolism** | | | | |
| AmdR | Zn2Cys6 + Fung Trans | Amide, omega amino acid and lactam utilization | *Aspergillus nidulans* | [132] |
| AreA | GATA | Regulation of nitrogen metabolism | *Aspergillus fumigatus* | [133] |
| AreB | GATA | Regulation of nitrogen metabolism | *Aspergillus nidulans* | [134] |
| ArgRII | Zn2Cys6 | Control of arginine metabolism | *Saccharomyces cerevisiae* | [135] |
| CpcA | bZIP | Regulatory network of amino acid biosynthesis | *Aspergillus nidulans* | [136] |
| LeuB | Zn2Cys6 | Regulation of *gdhA* and leucine biosynthesis | *Aspergillus nidulans* | [137] |
| MeaB | bZIP | Nitrogen metabolite repression | *Aspergillus nidulans* | [138] |
| NirA | Zn2Cys6 + Fung Trans | Nitrate utilization | *Aspergillus nidulans* | [139] |
| NmrA | NMRA | Nitrogen metabolite repression | *Aspergillus nidulans* | [140] |
| Nut1 | GATA | Regulation of nitrogen metabolism | *Magnaporthe oryzae* | [141] |
| PrnA | Zn2Cys6 + Fung Trans | Proline utilization | *Aspergillus nidulans* | [142] |
| TamA | Zn2Cys6 + Fung Trans | Co-activation of AreA | *Aspergillus nidulans* | [143] |
| UaY | Zn2Cys6 + Fung Trans | Purine utilization | *Aspergillus nidulans* | [144] |

| **Other regulators** | | | | |
| --- | --- | --- | --- | --- |
| AflR | Zn2Cys6 | Aflatoxin biosynthesis | *Aspergillus flavus* | [145] |
| HacA | bZIP | Activation of unfolded protein response | *Aspergillus oryzae* | [146] |
| HapB | CBF | CCAAT binding complex | *Aspergillus nidulans* | [147] |
| HapC | CBF | CCAAT binding complex | *Aspergillus nidulans* | [147] |
| HapE | CBF | CCAAT binding complex | *Aspergillus nidulans* | [147] |
| MetR | bZIP | Activation of sulphur metabolism | *Aspergillus nidulans* | [148] |
| PacC | C2H2 | pH regulation | *Aspergillus nidulans* | [149] |
| AnBH1 (PenR2) | HLH | Penicillin biosynthesis | *Aspergillus nidulans* | [150] |
| PrtT | Zn2Cys6 | Regulation of cellular protease encoding genes | *Aspergillus niger* | [21] |
| SreA | GATA | Iron homeostasis | *Aspergillus nidulans* | [151] |
| SrrA | HSF | His-Asp phosphorylation signaling | *Aspergillus nidulans* | [152] |
